# Comparative Odontography

**Published:** 1900-02

**Authors:** A. H. Thompson


### COMPARATIVE ODONTOGRAPHY.

BY A. H. THOMPSON, D.D.S.

Mr. President and Members of the Academy,—It gives me great pleasure to meet you all this evening. I appreciate your presence here, and I want to thank you for the compliment of electing me a member of the Academy, an honor which I esteem very highly, on account of the honorable standing that the Academy has and the valuable papers and discussions which all the profession esteem, and which I assure you are especially appreciated by those who, like myself, reside at a distance. I take great interest in reading the Transactions of the Academy. Many of my old friends are members, and I want to thank them for the honor of giving me the privilege of reading a paper before them to-night. I shall not attempt to instruct, but rather to entertain you, and for that purpose have prepared a few slides, which we will show you on the screen by way of illustrating, in a general way, the subject of Comparative Odontography. Dentists have a great deal to learn from the study of the teeth of the lower animals. These studies have not been used in our teaching in the curriculum of our colleges as much as they ought to be. This should be taught in an impressive way by the study of the teeth of the lower animals, just as the anatomist studies the parts and functions of animals in comparison with the human body; so I think that the teeth of the lower animals should be studied with this in view, for the better understanding of the teeth of man.

Without pretending to give anything comprehensive, I will begin with some of the lower forms in which teeth are first found. It struck me as remarkable that there should be found in an organism so low in the scale of animal life as the sea-urchin, an elaborate dental apparatus called "Aristotle's lantern." It is composed of five parts, which are arranged around a centre, forming a pyramid. There are five distinct alveoli, each one of which contains a tooth, which is quite like the incisors of the rodents,—having an enamel thick on one side and thin on the other; so that with use it is constantly kept on a sharp edge, wearing away more rapidly on one side than on the other. The teeth being in the centre of the

---

[1] Read before the Academy of Stomatology of Philadelphia, November 28, 1899.

test, they are protruded and retracted by means of the powerful muscles attached to the test on the inside. Being moved with powerful muscles, the teeth are used with a great deal of force for cutting and reducing food and for boring into rocks, and also shells in order to abstract the juices of the animal for food.

In the mollusks, too, we have a remarkable dental structure. Of course, the bivalves have no head or dental apparatus, but the single-shell mollusks have a head and a dental apparatus that are much complicated. The cuttle-fish, among the cephalopods, has a curious beak; but the dental apparatus is arranged on what is called the odontophore, or tongue, which is raised up over a cartilage, in order to throw it against the roof of the mouth; it uncoils itself, and as the teeth are worn new ones are brought into position for the purpose of cutting. In the snail these teeth are sometimes thrown forward and protruded, so that it is able to cut off plants and grass by means of this odontophore. The mollusks are provided with a great variety of teeth, all of which are classified and have particular names. One form is what is called the toxoglossa, which is found in the *conus* and some other of the sea-mollusks, and is of an arrow-shape, with a tube in the centre which contains poison, so that its bite is poisonous. There are a great many varieties of teeth in the various species of snails, but many of them merely have a hardened mass on the odontophore for the purpose of cutting plants. This is a very interesting field, as there is a great deal to be found out among mollusks in regard to their teeth. Some have but two or three teeth in a row, some have a dozen, some have twenty or thirty, some fifty, and some run up into the thousands. It offers special inducements to microscopists.

Coming to the vertebrates, we find a great deal of interest throughout this subkingdom. In the fishes, that class which is the lowest of the vertebrates, there are a great many interesting things to be found. The teeth of the fishes usually present the primitive form, the conical form of teeth, as shown in the pike, in which we have the hinge-teeth, which are pressed down by food passing over them, and then, springing back, seizes the prey and prevents its escape. The bony fishes that have this conical form of teeth are usually ankylosed to the jaw, except those which are attached by an elastic hinge. There are many types of the conical tooth, so that it is hardly necessary to go into details.

The teeth of the wolf-fish are not very sharp, but rounded for the purpose of crushing shells. It belongs to the same order as the pike. The sharks belong to the cartilaginous fishes, and their teeth are remarkable in that they are not ankylosed to the jaw, but are attached to a fibrous membrane. This fibrous membrane comes up from beneath the jaw and carries the teeth up over the edge of the jaw, where they stand erect. As they pull off or are naturally lost, they are being continually replaced by others on the membrane that comes up from the bottom of the jaw, from what is called the "thecal fold."

Among vertebrates, there are four principal methods of attachment of the teeth,—fibrous membrane, ankylosis, hinge, and socket implantation. The pike type represents two of these, and the shark represents that with the fibrous membrane. This type is found also in the rays, which are closely related to the sharks, as the teeth are brought up in the same way by means of a membrane within the jaw, although the teeth are differently formed, and are used for crushing shell-fish and substances of that sort taken into the mouth. There are a great many varieties of teeth among the rays, and they exhibit many remarkable forms. They are usually formed as a compressed cone which is depressed into a plate. Sometimes there are serrations on the plate, and sometimes there is quite a distinctive point in the centre, and occasionally a point on the side, a sort of triangular form.

Coming to the reptiles, we have some very interesting forms presented. The dental apparatus of the rattlesnake is very remarkable. The poison fang is erected, and is pushed forward by the superior maxillary bone. It lies back in the mucous membrane until the strike is made, and then it is pushed forward and raised. The remarkable form of the poison fang is to be observed in that it is rolled into a tube; the poison is not pushed through the pulp-cavity, but the fang is formed as if a canine tooth were taken and flattened out, and then it is curved over together, so that all the tissues are folded around the canal within,—the pulp, the dentine, and the enamel around the outside of the canal; and then it is covered again with a layer, perhaps, of cementum outside of that. All the tissues being folded in that way form the tube for the ejection of the poison. A remarkable thing in the formation of the poison fang is that the outlet of the poison is not directly on the point, but on the side of the point. From that suggestion came the idea of making the hypodermic needle,—first the point was put on the end, and then it was put on the side like the fang of a serpent, Nature having discovered that advantage before man did.

One of the most remarkable forms of reptiles is that of the turtles, which are without teeth proper, the masticating apparatus consisting of beaks like the bills of birds. Of course, there is some variety,—some are vegetable-eaters, which have the beaks built for crushing; others are carnivorous, and the beaks present a sharp edge for cutting flesh. The turtles are very interesting as showing some difference in the varieties of dental apparatus.

The crocodilia offer some interesting things in the study of the teeth, in that they are conically formed and show the true cone-form of tooth—that of the lowest form of vertebrates from which the mammalia, higher forms, were elaborated by duplication—as modification of the primitive cone. Alligators have these simple, conical teeth, which are implanted or imbedded in sockets. They are an advance on some of the other vertebrates, because in fishes the teeth are attached by ankylosis; in lizards attached either by ankylosis on the sides of the groove or implanted loosely in the groove. In the crocodiles the groove has a septum across between each tooth. We have the beginning of the true sockets in the crocodilia. These teeth are in continuous succession, as in the lower vertebrates; and they are pushed up as new teeth arise from the depths, so that they are developed in the socket of the tooth and carry the old teeth upward. These teeth are quite irregular in many forms. The teeth in the maxillary bones are small, and begin to rise to the large ninth tooth from the front, which is called the canine, but it is not related to the true canine. Then they decrease to smaller forms, then increase to the seventeenth tooth, and then decrease to the last, so there is some little system in the arrangement. There is some difference in the various types of the crocodilia. As between alligators and crocodiles there is little distinction. The alligator of the Mississippi Valley has the long tooth, or canine tooth, closed within the upper jaw in a sort of fossa, which sometimes becomes quite deep. This lower tooth may come up through the snout and project above. The crocodiles, however, have this tooth coming on the outside of the snout, and that marks a distinction between the species, and is one of the main differences. The teeth are almost alike as regards the size, shape, and in other respects.

Related forms are the caiman of South America and the gavail of India. The gavail is remarkable for having a spoon-shaped snout. It is very narrow and elongated, spreading out in a spoon-shaped form and being surrounded on the edges with sharp teeth. These all belong to the one family. One of the most remarkable discoveries of recent years among fossils is in regard to birds that had teeth,—the ichthyornis and hesperornis. One is quite a low form, in that the teeth are imbedded or implanted in distinct and continuous grooves, and in the other they are planted in the sockets. The teeth are of a low reptilian form, having a large base or bulbous root, by which they are attached. They are implanted in a ball, as it were, and this is set in the jaws. These forms are interesting as showing the transformation between the birds and reptiles. There are no living birds that have teeth, although sometimes they are found in bird embryos; and the discovery of these tooth-birds is quite remarkable, as showing a connection between the reptiles and the birds. And that takes us back to some of the fossil reptiles, as the fossil flying-lizard or pterodactyl, reptiles having teeth and extended wings, bat-like wings, but yet were distinctly reptilian. Some are quite small, but others had an expansion of twenty-five feet. These were found in fossil formations in the West, and are distinct species. Another beauty was the dinosaur, something like the kangaroo, having great hind-legs and short forelegs. Some of these were large, standing up as high as a giraffe, but being organized a great deal like a kangaroo, with great heavy hind-feet, used in locomotion, and small forefeet. Some of the other forms are remarkable. They are ugly-looking beasts. Some of these forms were very likely herbivorous, being plant-eaters, some were flesh-eaters. There were some other remarkable forms, some having great crests or scales along the vertebral region, probably armed for the purpose of protection against the attacks of other animals.

Coming to the mammals, we find in the lower forms that the lowest mammals present the type of oral armature which is found in the lowest forms of fishes and of reptiles, that is, the horny beak instead of the teeth. This is found in the duck-bill mole of Australia, whose jaws are covered with a horn-like sheath, for the purpose, as in the duck, of straining food through the water. It used to be supposed they had no teeth, but recent specimens show them to have some calcareous teeth in the back part of the mouth. A curious fact is that teeth have been developed, aborted, and then lost, and the horny beak developed on the jaws afterwards, showing that they descended from a form which originally was possessed of true teeth. It is a very interesting form, bearing some resemblance to the duck on the one hand, and the mole on the other.

In the kangaroo, the lower incisors project and close within the upper incisors; so we have formed almost a scissor-like edge at that point. The remarkable thing about the jaw of the kangaroo is that the halves of the lower jaws are somewhat flexible, and there is quite an edge along the mesial line of the incisors, by which they shear grass from the ground; and in Australia the sheep have starved by thousands on account of the shaving off of the pastures by kangaroos. It is quite unique that the jaw is arranged so that the teeth can be moved against each other by a lateral motion.

Coming now to the higher form of mammals, we notice first the teeth of the carnivora, which are highly specialized and belong to an extreme degree of organization. We have plant-eaters at one end and at the other end the flesh-eaters,—the carnivora and herbivora. The cats proper have the jaws and the teeth arranged so that they can use them for the purpose of cutting and dividing flesh. The jaw of the cat is articulated, so that there is but the vertical motion, for cutting flesh, and the sharp-bladed teeth pass each other with a shear-like motion. There are no tubercular teeth. Mastication is not provided for by any tubercular forms of teeth until we advance towards the herbivorous types, when it is observed that the teeth and jaws again bear a strong relationship to each other. On account of the vertical motion of the jaws in the carnivora, the teeth are developed in the direction of the greatest use, that is, vertically. As we pass from the cats towards the more omnivorous forms, we begin to have tubercular teeth, which indicate a more mixed diet. As we advance to the bear, we have still more tubercular teeth, which are used for a still more omnivorous diet, because the bear and man are a good deal alike in regard to the forms of molars. The tubercular molars are used especially for a mixed diet. In the dog we have more tubercular teeth, and a larger number in the cats, who have but few teeth, most of the molars being aborted. In the dog we have larger molars and increase in the tubercular form of teeth, which indicates a more mixed or omnivorous diet. In the dog it is interesting to observe the changes that take place in regard to their organization. In the pug-dogs many of the teeth are aborted, and those that remain are very much crowded. In the hounds, which have long jaws, the teeth are the full number, and sometimes there are extra molars. There are more molars in the jaws of dogs that have the long jaws. This is interesting in connection with the different types. The upper molar of the opossum is triangular. It is the survival of a very primitive type of molar. The triangular molar is found far back in ancient times and is a primitive form of the upper molar. We sometimes have reversions to that type in man, in which we observe a triangular molar above, the fourth cusp being sometimes absent. This triangular molar is retained in the opossums and others. In many forms the fourth cusp has been added to give a larger masticating area. In the raccoon we have a wide tubercular form of molar, and in the skunk and in some such forms the molars are carnivorous and insectivorous to a degree. They have molars, also, for grinding purposes. Many interesting things have been discovered among fossil animals. In what is called the great "sabre-tooth" tiger we observe especially the elongated canine tooth which projected far down below the lower jaw and caused a flange-like development of the rim of the lower jaw, as if making a sheath for its projection. This tooth was developed to such an extent that it is a question as to how it could have been used when the mouth was closed, and whether the animal could have seized anything with the canine tooth; so it is supposed it must have been used with the mouth closed. In South America and in the cretaceous beds of the West there have been found specimens in which there was no flange on the lower jaw; we suppose they employed these teeth with the mouth closed, ripping up their prey on the side. In the fossil forms in South America, skulls have been found in which the lower jaws were caught on the canines and the animal starved to death. That would make a limit to the evolution of that form.

Coming to the opposite extreme, from the carnivoræ, we have the plant-eaters which subsist on vegetable matter. The herbivora eat the plants and the carnivora eat the plant-eaters. There are no teeth in the upper jaw in the incisal region, the lower incisors closing against the gums, as in the cow, goat, sheep, and all the ruminants. It is an interesting thing to note how, in the ruminants, the motions of the mandible and the development of the teeth in relation to the motion of the jaw are worked out. That is, we have the extreme lateral movement for the purpose of grinding; and, as a consequence, in response to this lateral movement, the molar teeth were developed laterally and in the direction of the greatest resistance. The teeth of the herbivoræ develop in such a way as to make them most useful in the reduction of their food; for instance, the enamel, dentine, and cementum are arranged so that in wearing, they being of different densities, there is a constantly rough surface presented, which insures a perfect grinding implement. The enamel being hardest stands highest, the dentine next, and the cementum is worn out most, being the softest. The lateral movement of the jaw is necessary for the purpose of masticating the food, because plants require extensive manipulation. It is interesting, also, to observe that in each of the families of plant-eaters there is a distinct type of molar; for instance, the cow has a distinct form, also the deer family, the giraffe, the camel, the horse, rhinoceros, hippopotamus, and so on. Each of these families has a distinct type of molar pattern which comparative anatomists come to recognize as belonging to it, and it is diagnostic of that family.

In the cow we observe the type of the molar pattern, which is distinctive; there is absence, also, of the incisors in the upper jaw, their place being taken by the gum-pad, which receives the impact of the lower incisors. There are a few anomalous forms among the ruminants, as the musk-deer, in which there are quite long canine teeth, probably for battle purposes, the horns being absent, which is quite unique in this family, these canines projecting down far below the jaws. These are found, also, in a few others of the deer family.

The horse has a distinct molar type which is characteristic of the horse in all branches of the family. We do not quite understand why the ruminants—the cow, deer, etc.—have no incisors above, and the horse, which subsists on the same food, does have them. It is one of the evolutions of Nature that we do not comprehend. The horse is a non-ruminant and has a distinct set of upper incisors, and the ruminants have not. The phylogeny or evolution of the horse has been well worked out. The earliest horse had five toes. Then in later forms reduced to three toes. Then the present singled-toed horse was evolved, whose ancestors have been found in the cretaceous beds of the West. It is one of the triumphs of evolution that the phylogeny of the horse has been completely made out. In all other families there are many missing links, so that we cannot tell definitely as to all the stages of their evolution; but in regard to the horse its history has been worked out wonderfully and thoroughly, because the stages have all been found and the specimens are to be seen. I think these are found mostly to-day in Professor Cope's collection. The advances that have been made and what science can do are wonderful.

There are more rodents to-day than any other living order of mammals. They are distinctly known by their long chisel-like incisors, which grow from the upper and the lower jaws and meet together at the edges. This is a type of teeth which grow from persistent pulps and grow continuously, so that they need to be worn off in order to compensate for the continuous growth. The enamel is thicker on the outer side than it is on the inside, wearing the end to an edge. There are many kinds of rodents,—as the squirrel, the beaver, the ground hog, rats and mice, prairie dogs, etc. The beaver has very strong incisors for cutting sticks for building their well-known dams. Sometimes, if an accident happens and one of these teeth is broken, the opposing tooth continues to grow, interfering with mastication, causing the death of the animal by growing in a circle, and sometimes penetrating the head. An abscess from an accident may interfere with the growth of the tooth and throw it to one side, causing it to grow without opposition. Sometimes there is an accident to an upper incisor, so that the lower incisor continues to grow and prevents the animal from masticating its food and it dies from starvation, showing that the wearing of the incisor compensates for the continuous growth. In menageries, beavers have to undergo a dental operation sometimes to have the incisors reduced or filed off. They have to be watched in menageries for that purpose.

We come next to the mammoth, the great elephant of the north of Asia and of America, which was the progenitor of the elephants of to-day. The mammoth has been found in the flesh frozen in the ice of North Siberia. A fine specimen is in the Museum of St. Petersburg, the body of which was found frozen in the ice at the mouth of the river Lena, and for years the natives fed their dogs on the flesh thereof. After a time the authorities heard of it, and managed to rescue some of the skin with the hair on, and this is preserved with the skeleton to-day in the same museum. Although the animal is extinct, it was a true elephant, probably the progenitor of many elephants which are also now extinct. The tusks were enormous, extending out as far as twenty feet. These tusks have been found in great quantities in Siberia and the islands and shoals of the Arctic Ocean. Most of the ivory used a hundred years ago came from this great deposit of fossil ivory in Northern Siberia, or was dredged up from the Arctic Ocean, showing that the animals existed in great numbers at an early period. In the mastodon the teeth are somewhat different from those of the elephants proper, in that they have ridges and nipple-like protuberances on the occlusal surface, while the elephants' molars are on a plane. The mastodon differed from the elephant, also, in that he had two small tusks projecting forward from the lower jaw in addition to those of the upper jaw, which were like the tusks of the elephant, which project from the upper jaw. Another form was that of the dinotherium, which had tusks curving down like a sort of pick, with which he dug the plants from the bottom of the rivers for his food, the upper tusks being entirely aborted.

The enamel plates of the molars are bound together by means of cement, the dentine being within and the cementum without. The form of molar has a relationship to the movement of the jaw in mastication. This movement of the jaw we find is anteroposterior, not lateral; so the enamel plates are arranged transversely to the movement of the jaw, so as to resist and catch the food in the process of the mastication.

The method of the eruption of the teeth of the elephant is quite peculiar, in that the teeth succeed each other horizontally, the tooth passing down through the groove, and not vertically, as with most mammals. In the menageries they have considerable difficulty with the teeth of elephants on account of the lack of wear. In their native wilds they pick up a great deal of gravel and sand, that wears the teeth down and keeps them in order for the purposes of mastication; therefore the keepers are obliged to add sand and gravel to their food in order to keep them worn down in the natural way. The tooth of the mastodon is the original form from which the other elephant teeth were developed. The dinotherium also had molars like the mastodon.

We come now to the quadrumana. In the lowest form, the lemur, the lower incisors project horizontally forward and close against or between the incisors above. This type bears little resemblance to the advanced form of the higher types; but in the lemur there are interesting specimens observed. In some forms the incisors are separated, and the lower incisors project horizontally and pass between the upper incisors, so as to form a comb, and on account of that the animal uses its teeth to a considerable extent as a comb for dressing its fur.

In one American monkey, the howler, the large lower jaw is developed and spread out by the exceedingly large larynx of the animal. Its howls can be heard for miles in the South American woods. The neck is enlarged and has an abnormal appearance. The American monkey differs from the European in having three premolars. It has thirty-six teeth instead of thirty-two. The European or Old World monkeys have but two premolars, like man. There are three in the American monkeys. In some forms of the

American monkeys there are thirty-two teeth, the third molars being absent.

The baboon, as all of the Old World monkeys, have the same dental formula as man, having thirty-two teeth,—two premolars and three molars. The baboon is remarkable for the form of the first lower premolar, the crown and side of the root being raised to a cutting-edge, and the long upper canines closing past them, making a very formidable cutting appliance; there are distinct edges on the upper canine and on the lower premolar. The molar teeth are not as well developed, for we have not yet gotten to the oblique ridge found in some of the higher quadrumana.

The orang-outang is one of the higher types of monkey. It has the same dental formula as man, and its teeth resemble the human teeth to a remarkable degree, except in being enlarged,—the canines protruding very much, and the presence of the diastema in front of the canine into which the lower canine closes. There is much prognathism in this animal. It has the receding chin, like the idiot, which is observed sometimes in some of the lower races.

In the gorilla, which is the highest of the apes, we have a still more advanced type,—that is, nearer to the human form in many parts of its organism than any other lower animal. The denture is not quite as pronounced in its human type as that of the chimpanzee, because in the latter the third molar begins to be reduced; but in the gorilla it is functional, and quite as large as the other molars. We have the oblique ridge quite well developed and distinctly typical. The bicuspids are well developed, and the transverse ridge connecting the cusps is very strong.

There is a gradual reduction, of course, from the gorilla up to the man, in the evolution of the molars; the type of the teeth is still there, and there is no mistake about the relationship. In the lower forms we have not got the oblique ridge; but in the gorilla this oblique ridge appears, as in most of the higher apes.

In the fossil skulls found in Europe some years ago the features were of a distinctly low type, and had many distinctive apelike characteristics. There were the prominent superciliary ridges, the pronounced prognathism, the receding forehead, the chin receding (a very distinct apelike feature), as in the receding chin of the chimpanzee or modern monkey.

In the Sky man there is the apelike form, the later prominence of the chin being an indication of the elevation of the race. So in many of the lower forms we find this receding chin, which is a distinct Simian-like feature. Some of the teeth were of these lower types, showing the full forms of the molars, and also the fifth cusp on the second lower molar, which is an apelike feature.

In prognathous lower races the third molar shows anteriorly to the ramus of the lower jaw. In the European and higher races the third molar is concealed. There is a pushing forward, not only of the anterior part of the jaw, but of the entire jaw,—prognathism of the entire jaw.

The Australian is one of the lowest living races. We have in the second lower molar the fifth cusp, which is a distinct apelike feature; the third molar is quite distinct in form. The chin is somewhat prominent, but not as much so as in some of the higher races.

The Hawaiian is not so low as the Australian, but shows a distinct type. The prognathism and the receding chin are very typical, and the lower molar shows the fifth cusp.

I hope my rapid review of this valuable subject has been interesting to you, and that it will stir up your interest. There is a great deal to be learned from the subject of comparative dental anatomy as a means of illuminating the important science of human odontology, and therefore we contend that it should be much more extensively studied and taught in our colleges than has been heretofore done.

